# A rare case of arrhythmogenic right ventricular cardiomyopathy associated with *LAMA2* mutation: A case report and literature review

**DOI:** 10.3389/fmed.2022.922347

**Published:** 2022-07-18

**Authors:** Yue Wang, Yibing Fang, Dan Zhang, Yifei Li, Shuhua Luo

**Affiliations:** ^1^Department of Cardiovascular Surgery, West China Hospital, Sichuan University, Chengdu, China; ^2^Department of Cardiovascular Surgery, Southwest Hospital, Army Medical University, Chongqing, China; ^3^Key Laboratory of Medical Electrophysiology, Ministry of Education, Medical Electrophysiological Key Laboratory of Sichuan Province, Institute of Cardiovascular Research, Southwest Medical University, Luzhou, China; ^4^Key Laboratory of Birth Defects and Related Diseases of Women and Children of MOE, Department of Pediatrics, West China Second University Hospital, Sichuan University, Chengdu, China

**Keywords:** *LAMA2*, ARVC, iPSC, case report, literature review

## Abstract

**Background:**

Arrhythmogenic right ventricular cardiomyopathy (ARVC) is a heritable heart muscle disorder that predominantly affects the right ventricle. Mutations in genes that encode components of desmosomes, the adhesive junctions that connect cardiomyocytes, are the predominant cause of ARVC. A case with novel heterozygous mutation in the *LAMA2* gene is reported here. The protein encoded by *LAMA2* gene is the α2 chain of laminin-211 protein, which establishes a stable relationship between the muscle fiber membrane and the extracellular matrix. We explored the potential mechanism and the relationship between the mutation and ARVC.

**Case Presentation:**

At the age of 8, the patient developed syncope and palpitation after exercise. Dynamic electrocardiogram recorded continuous premature ventricular beats, and MRI showed the right ventricle was significantly enlarged and there were many localized distensions at the edge of the right ventricular wall. The patient was diagnosed with ARVC and received heart transplantation at the age of 14 due to severe heart dysfunction. The myocardial histological pathological staining revealed a large amount of fibrosis and adipose migration. Whole exome sequencing (WES) identified the heterozygous mutation in the *LAMA2* gene [NM_000426.3: c.8842G > A (p.G2948S)]. This is the first report of these variants. Analysis was performed on genetic disorders to reveal splice site changes and damage to protein structure. *LAMA2 p.G2948S* predicted unstable protein structure and impaired function. Induced pluripotent stem cell-derived cardiomyocytes (iPSC-CMs) were established. RNA-seq and the western blot were performed on IPSC-CMs to explore the ARVC-related signaling pathway.

**Conclusion:**

This is the first case report to describe an ARVC phenotype in patients possessing a novel *LAMA2 c.8842G* > *A* (*p.G2948S*) mutation. Our results aid in understanding of the pathogenesis of ARVC. The molecular mechanism of *LAMA2* leading to ARVC disease still needs further study.

## Introduction

Arrhythmogenic right ventricular cardiomyopathy is an inherited cardiomyopathy characterized by the fibrofatty replacement of the myocardium predominantly in the right ventricle. Progressive loss of right ventricular myocardium and its replacement by fibrofatty tissue is the pathological hallmark of the disease (1, 2). Mutations in the genes encoding desmosome complex, which is important in regulating cell-to-cell adhesion and maintain cellular integrity, may affect protein structure of desmosome complex. The degradation of desmosome has been believed to be involved in the pathogenesis of ARVC. Until now, most of the ARVC-related genetic variants have been located in the desmosome complex. In addition, other non-desmosome genes, which participate in mediating cellular adhesion and intercellular connection, would also contribute to induce ARVC, and increasing numbers of mutations have been underlined. In detail, the reported genetic mutations of ARVC encoding proteins cover a large diverse range of biological functions, including cytoskeletal architecture formation, calcium handling, sodium transport, and cytokine signaling (2). Nevertheless, negative genetic results have been found in 35–50% of patients, suggesting that there may be unknown genes involved in the pathogenesis of ARVC (3, 4).

The *LAMA2* gene encodes the α2 chain of laminin-211 protein, which establishes a stable relationship between the muscle fiber membrane and the extracellular matrix (ECM). Laminin 211 is dominantly expressed in the basement membrane of myocytes and Schwann cells. The mutations of *LAMA2* have been proved to be involved in the initial of congenital muscular dystrophy type 1a (MDC1A) and limb girdle muscular dystrophy (LGMD) (5, 6). Laminin-α2 has also been proved to be expressed in other organ tissues, such as the heart (7). However, current evidence suggests that only a small proportion of patients with MDC1A is associated with cardiac-related clinical manifestations, and the correlation between *LAMA2* mutation and ARVC remains unknown (8, 9). And only one record from the ClinicalVar database demonstrated *LAMA2* might be related with primary dilated cardiomyopathy. Induced pluripotent stem cell-derived cardiomyocytes (iPSC-CMs) have been used as a fully mimic platform to study the biological and molecular mechanism of any identified uncertain genetic variant. Several cell lines had been established to demonstrate the essential changes on ARVC-related genetic mutations. Thus, we attempt to explore the relationship between *LAMA2* mutation and ARVC *via* iPSC-CMs.

Herein, we report a rare case with ARVC carrying a novel heterozygous mutation in the *LAMA2* gene. This should be the first case report of ARVC due to a *LAMA2* mutation, and we also established the first iPSC line of *LAMA2* associated with ARVC, which would be differentiation into cardiomyocytes to address the potential activated ARVC-related pathogenic signaling pathways.

## Case presentation

### Ethics compliance

The parents of the patient provided written consent for participating in the study carried out at the West China Hospital, Sichuan University, China. This research study was approved by the Ethics Committee of West China Second University Hospital, Sichuan University, China (2014–034).

### Clinical presentation and outcomes

**FIGURE 1:**
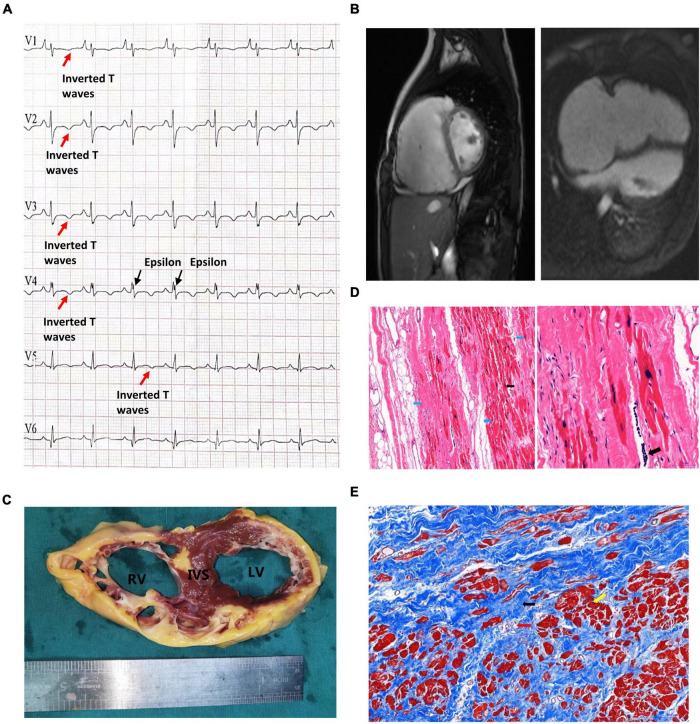
Clinical presentation of the proband. **(A)** The patient’s preoperative ECG showed inverted T waves in lead V1, V2, V3, V4, and V5, with typical Epsilon waves visible in V4. **(B)** Preoperative cardiac MRI of the patient indicated that the right atrium was significantly enlarged, the right ventricular wall was thinner, the right ventricular wall edge was localized in many places, and the amplitude of myocardial contraction of the right ventricle was significantly reduced. **(C)** The myocardium was replaced by extensive fibrous adipose tissue in the wall of the right ventricle (RV), the right ventricle was bag like and thinly dilated, part of the ventricular septum (IVS) was adipose, and part of the myocardium in the anterior and lateral wall of the left ventricle (LV) was replaced by adipose fibers. **(D)** HE staining of the myocardium of the right ventricle: Left: the blue arrow indicates the adipose tissue, and the myocardium of the right ventricle is surrounded by the adipose tissue and divided into cords. The black arrow shows abnormal hypertrophic cardiomyocytes with large, dark-stained nuclei and oddly shaped nuclei, broken muscle fibers in the cytoplasm of the cardiomyocytes (magnified 100×). Right: The black arrow indicates the presence of inflammatory cell infiltration between cardiomyocytes at magnified 200×. **(E)** Masson staining of the myocardium of the right ventricle. Note that the myocardium (the yellow arrow) is surrounded by large amount of fibrous adipocytes (the black arrow) and divided into cords or islands. The collagenous fiber bundles around small blood vessels (red arrows) are increased.

This proband was a male who had been admitted to our hospital due to aggressive fatigue, chest tightness, and fainting for 2 years. He experienced significant chest pain and palpitations shortly before syncope, with a decrease in tolerance of daily activities. A pale face, dull heart sound, and third-degree systolic murmur in the fourth intercostal space were observed. Results of physical examination of the respiratory and nervous systems were negative. The parents of the patient declined any cardiovascular symptoms. Myocarditis has been excluded at first due to the patient, and his parents declined any no related nearby infection history. Furthermore, the patient declined any symptoms of muscular dystrophy. Then, any potential virus antibodies were negative after serum analysis. Electrocardiogram (ECG) demonstrated inversion of T waves in leads V1–V5, and epsilon waves were visible in some leads (Figure 1A). Holter presented 5,443 premature multisource ventricular beats in 24 h, with unequal pairing intervals, ventricular fusion wave, and insertion ventricular premature beats. MRI identified enlargement of the right ventricle, the thinner right ventricular wall, and localized distensions at the edge of the right ventricular wall. The myocardial contraction of the right ventricle was significantly reduced, and ejection fraction of the right ventricle was 15%. Right ventricular end-diastolic volume index = 142 ml/m^2^ (Figure 1B), so that, according to Task Force Criteria (10), this patient received a clinical diagnosis of ARVC based on his clinical manifestation, classic ECG presentation, and a cardiac phenomenon recorded by MRI.

Unfortunately, due to severe and irreversible heart dysfunction, the patient received heart transplantation at the age of 14. The examination of the heart tissue revealed generally adipose infiltration in the interventricular septum and partial fiber-adipose infiltration in the myocardial tissue of the posterior wall of the left ventricle (Figure 1C). The wall of the right ventricle is extensively fiber-adipose, and only a small amount of myocardial tissue remains. The whole right ventricle is obviously dilated. And the myocardial histological pathological staining revealed a large amount of fibrosis and adipose migration (Figures 1D,E).

After the heart transplantation, the patient had a good quality of life and was followed for 3 years.

## Molecular results

A peripheral blood sample was obtained from the patients in an EDTA anticoagulant blood sample tube stored at 4°C for less than 6 h, and DNA was extracted. Protein-coding exome enrichment was performed using the Agilent SureSelect Human All Exon V6. The whole exon sequencing (WES) of the patient and his parents were performed using the NovaSeq 6000 platform (Illumina, San Diego, CA, United States), and the raw data were processed using FastP to remove adapters and filter out low-quality reads. Paired-end reads were aligned with the ENSEMBL GRCh38/hg38 reference genome using the BWA software (version 0.7.12-r1039). Then, the SAMtools software (Version 1.3.1) was used to compare and order the results, and Sambamba (Version 0.7.1) was used to mark the repeated reads. Variant annotation was performed in accordance with database-sourced minor allele frequencies (MAFs) and practical guidelines on pathogenicity issued by the American College of Medical Genetics. The annotation of MAFs was performed based on the 1,000 Genomes, dbSNP, ESP, ExAC, Provean, Sift, Polypen2_hdiv, Polypen2_hvar, and Chigene in-house MAF databases using R software (R Foundation for Statistical Computing, Vienna, Austria). The sequencing data have been deposited in the GSA database.

**FIGURE 2:**
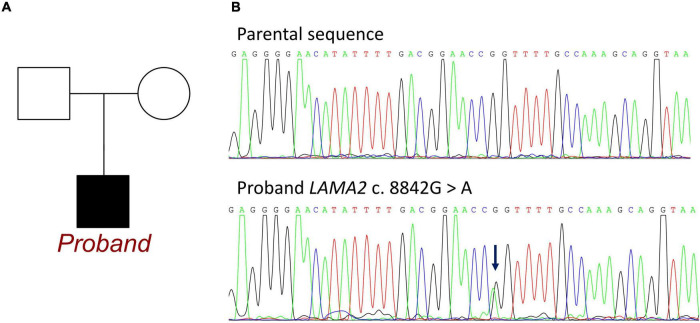
Information of *LAMA2* c.8842G > A in the family of the proband. **(A)** Family map of gene mutation. **(B)** Sequencing of mutations. Blue arrow: The normal group showed single peak of base G, while the patient showed heterozygous state of base G peak and A peak.

Whole exon sequencing identified *de novo* heterozygous mutations in the *LAMA2* gene at c.8842G > A, which has not been reported in any publication. This mutation was absent from the patients’ parents (Figure 2). This mutation site could be retrieved in 1000G and the ExAC database. There were 3 records of allele carriers in 1000G and 46 records of allele carriers in ExAC. Its clinical associations were found in congenital muscular dystrophy (RCV000764636.1), *LAMA2*-related dystrophy (RCV001086813.1), and primary dilated cardiomyopathy (RCV001293224.1). All the records were submitted by a gene sequencing company, and no available publication demonstrated the molecular mechanism and provided a detailed clinical report. Until now, no research has declaimed a clear relationship between *LAMA2 c.8842G* > *A* and ARVC. According to the American College of Medical Genetics, the variant has uncertain pathogenicity. SIFT, Polyphen_HumDiv, Polyphen_HumVar, and Mutation Taster were used for mutation locus screening. Analysis performed with MutationTaster revealed that this mutation was considered disease causing due to amino acid sequence changes, protein features affected, and splice site changes (probability = 0.999). The PolyPhen tool predicted protein structural and functional damaging for p.G2948S (Polyphen_HumDiv score = 1.000, the Polyphen_HumVar score = 0.999). The SIFT prediction on the protein batch demonstrated a damaging change as a score of 0.006. And the PROVEAN prediction on the protein batch revealed a deleterious change as a score of −4.34. All analyses were based on the P24043 FASTA sequence of the G2948S variant. However, it failed to identify any other known ARVC-related gene mutations both in exons and introns.

To understand the molecular architecture of the human *LAMA2* gene, we performed comparative modeling using the SWISS-MODEL^1^. We estimated the change in the free energy of the model using the mutation cut-off scanning matrix (mCSM) method^2^, the Site Directed Mutator (SDM)^3^, and the DyanMut method, which can be used to analyze and visualize protein dynamics by sampling conformations and assess the impact of mutations on protein dynamics and stability^4^. To assess the impacts of mutations on the stability of *LAMA2*. We also used the DUET server^5^ that integrates mCSM and SDM to improve the overall prediction accuracy of the mutations under consideration. The signature vector that was ultimately generated was employed to train the predictive classification and the regression model used to calculate the change induced by mutations in terms of Gibbs folding free energy (ΔΔG).

**FIGURE 3:**
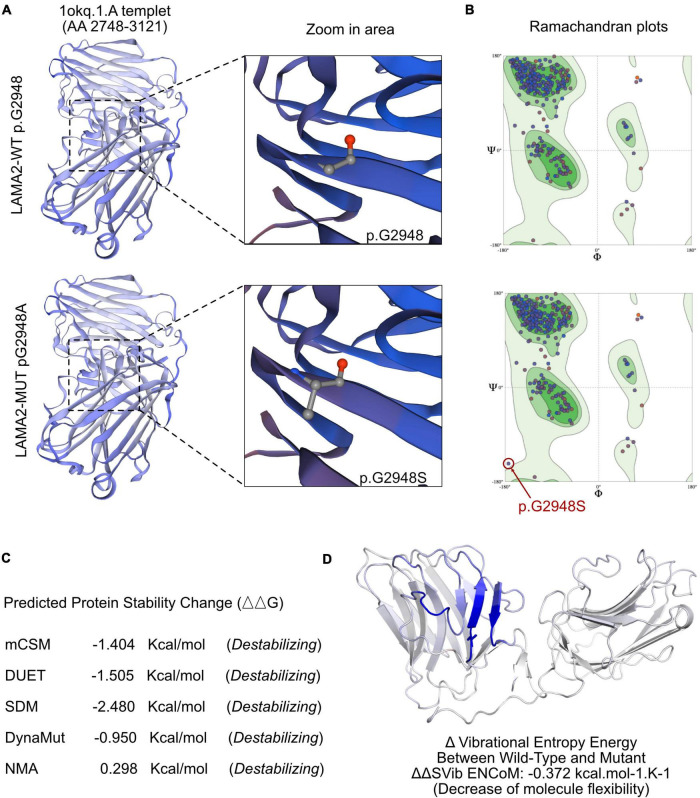
The effects of *LAMA2* c.8842G > A mutation on the molecular structure of the protein. **(A)** Individual crystal structures of wild type and G2948S according to the 1okq.1.A model template. **(B)** Ramachandran plots of amino acid with wild type and p.G2948A. **(C)** The comparisons of free energy on crystal structure of the wild type sequence and the p.G2948A variant. **(D)** The structural changes due to the mutation of *LAMA2* G2948S.

The SWISS-MODEL tool was used to analyze stability after amino acid changes. Ramachandran plots indicated that amino acid positions were altered (Figure 3A). Rebuilding molecular structure based on a 1okq.1.A template resulted in residue changes between Gly and Alaat 2948 (Figure 3B). Five types of calculation methods all demonstrated significant destabilizing change (DynaMut −0.950 Kcal/mol; NMA-Based Predictions, 0.298 Kcal/mol; mCSM, −1.404 Kcal/mol; DUET, −1.505 Kcal/mol; SDM, −2.480 Kcal/mol; Figure 3C). Besides, change in vibrational entropy energy between wild-type and mutant Figure 3D demonstrated a decrease of molecule flexibility as −0.372 kcal/mol K. The blue-colored amino acids sequence according to the vibrational entropy change upon mutation represented a rigidification of the structure.

### Induced pluripotent stem cell reprogramming and validation

Peripheral blood mononuclear cells (PBMCs) were isolated from 20 ml of blood obtained from the patient with *LAMA2 c.8842G* > *A*. And the wild-type control was obtained from a healthy donor, who was confirmed without any pathogenic variant, including *LAMA2*, by another WES. The third-generation PBMCs cells with good growth status were induced to differentiate into pluripotent stem cells. The PBMCs cells were cultured in a StemPro-34 complete medium containing cytokines IL-3, IL-6, SCF, and FLT-3 for 4 days. Sendai virus containing four reprogramming transcription factors [OCT3/4 (MOI = 3), SOX2 (MOI = 5), KLF4 (MOI = 3), and C-MYC (MOI = 5)] was added into the StemPro-34 complete culture medium (provided with a Sendai virus reprogramming kit) and cultured in a cell incubator for 24 h. For the first 3 days after transfection, the cells were maintained in the StemPro-34 complete culture medium without virus. Sendai virus-infected PBMCs were transferred to Matrigel-coated 12-well plate containing an E8 medium. iPSCs, (WCHi002-A) were manually picked at around day 20 post transfection and plated onto a Matrigel-coated 12- well plate. iPSCs differentiated to cardiomyocytes by modulating WNT signaling with CHIR99021 and IWR-1. iPSC-CMs were enriched by culture in lactate-containing media. Spontaneous beating activity can be observed in iPSC-CMs and the expression of myosin light chain 2 (MYL2) and cardiac troponin T (CTNT) can be detected by immunofluorescent staining.

**FIGURE 4:**
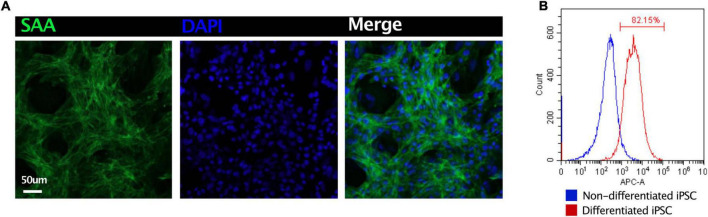
Quality control of iPSC-derived CMs. **(A)** Immunostaining of sarcomeric alpha-actinin (SAA) demonstrated the high ratio of CMs. **(B)** Flow cytometry revealed 82.5% iPSC had been differentiated into CMs.

Induced pluripotent stem cell-derived CMs were fixed in 4% paraformaldehyde overnight. The cells were incubated in a blocking medium (PBS containing 5% donkey serum, 0.2% Triton X-100) at 4°C overnight. For immunostaining, the sections were incubated with primary antibodies at 4°C overnight and secondary antibodies for 2 h. The primary antibodies used were as follows: sarcomeric alpha-actinin (1:200, Abcam, ab137346). After washing with a blocking buffer, the slices were incubated with optimal secondary antibodies with DAPI at room temperature for 2 h. After washing, the samples were imaged using an Olympus FV1000 confocal microscope (Figure 4A). Flow cytometry had been applied to demonstrate the purity of iPSC-CMs. Non-differentiated iPSC and differentiated iPSC were stained with cardiac troponin I (1:200, Abcam, ab56357). More than 80% of CMs had been identified (Figure 4B).

**FIGURE 5:**
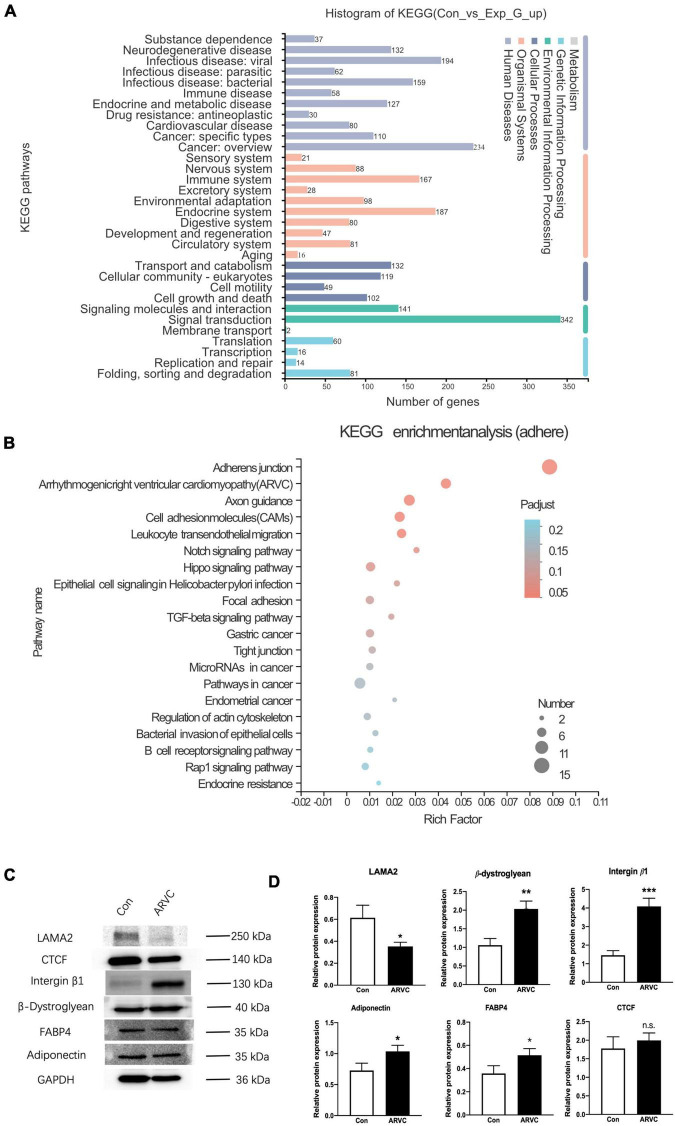
The analysis of iPSC-derived CMs indicated an ARVC-associated phenotype. **(A)** Histogram of KEGG signaling pathway classification. **(B)** KEGG enrichment analysis of the adherent signaling pathway. **(C)** Western blot results showed the protein expressions of LAMA2, CTCF, Integrin-β1, β-Dystroglycan, FABP4, and Adiponectin in myocardium cells of the control group and the ARVC group, with GAPDH as internal reference. **(D)** Histogram shows the protein expression differences of LAMA2, CTCF, Integrin-β1, β-Dystroglycan, FABP4, and Adiponectin between the control group and the ARVC group (N.S. The difference is meaningless; *p < 0.05; **p < 0.01; ***p < 0.001).

The TRIzol method was used to extract total RNA from iPSC-CMs of ARVC (*n* = 3) and WT (*n* = 3) groups, and the DNase I method was used to remove DNA in the samples. An Illumina HiSeq XTEN/NovaSeq 6000 sequencing platform was used for high-throughput sequencing. Transcript abundance was determined by TopHat alignment followed by HTSeq-Count and statistical analysis by DESeq2. Enrichment analysis of differentially expressed genes (DEGs) was implemented by the clusterProfiler R package, in which gene length bias was corrected. KEGG pathways with corrected *P*-value less than 0.05 were considered significantly enriched by DEGs. According to the established pathophysiological characteristics of ARVC, five enrichment sets were established, including fibrosis, adipogenesis, apoptosis, intracellular calcium, and cellular connectivity (Figure 5A). The KEGG pathways of fibrosis were enriched in PI3K-Akt signaling and regulation of actin cytoskeleton (Supplementary Figure 1). The enrichment analysis for lipid metabolism revealed a general alternation of lipid metabolic dysfunction (Supplementary Figure 2). In addition, apoptosis had been recorded by KEGG enrichment (Supplementary Figure 3). Most important, ARVC was characterized as misleading calcium handling and cellular connection disorders. After KEGG analysis based on DEGs, the significant calcium signaling pathway and cGMP-PKG signaling pathway were identified (Supplementary Figure 4). Furthermore, the changes in adherens junction, cell adhesion molecules, and the hippo signaling pathway had been proved to be participated in pathological remodeling of cardiomyocyte due to *LAMA2* mutation. And the enrichment of ARVC was definitely identified among all DEGs (Figure 5B).

We queried all possible related signaling pathways of ARVC through KEGG website^6^, and found the *LAMA2* and its related proteins integrin beta 1 (ITGB1) and β-muscle dystroglycan 1 (β-DG) are involved in the ARVC signaling pathway. The protein expressions of LAMA2, ITGB1, and β-DG were further detected by the Western blot. Normal healthy human PBMCs were successfully reprogrammed and inversely differentiated into iPSCs according to the above scheme, and were induced into cardiomyocytes as the control group. Both groups were continuously cultured in a myocardial maintenance medium for 50 days. Differentiated iPSC-CMs were lysed in RIPA lysis buffer system (Santa Cruz Biotechnology, sc-24948) with Mini Protease Inhibitor Cocktail Tablets (cOmplete, 4693124001). Total protein concentrations were normalized using BCA analysis (Life Technologies, 23227). After boiling with a 4X loading buffer for 5 min, 20-μl cell lysate of each sample was separated on a 10% gel, transferred to a PDVF membrane, and blocked by 4% BSA/TBST. Primary antibodies of LAMA2 (1:2,000, Abcam, ab140482), β-DG (1:2,000, Abcam, ab3125), ITGB1 (1:2,000, Abcam, ab52971), Adiponectin (1:2,000, Abcam, ab181281), FABP4 (1:2,000, Abcam, ab92501), CTGF (1:2,000, Abcam, ab209780), and GAPDH (1:2,000, ProteinTech, 60004-1-lg). The data were analyzed with GraphPad Prism 8.0 statistical software by *t*-test. LAMA2 protein expression in the ARVC group demonstrated significant decreased (*p* = 0.02), ITGB1 (*p* = 0.001), and β-DG (*p* = 0.004) protein expressions were significantly upregulated (Figures 5C,D).

## Discussion

This study demonstrated a rare clinical case with a *LAMA2* genetic heterozygous mutation (c.8842G > A, p.G2948S). The patient was initially diagnosed with ARVC based on clinical presentation and radiological images examinations according to Task Force Criteria. After several years of medication administration, the irreversible heart failure and aggressive cardiac pathological remodeling led this patient to receive a heart transplantation at the age of 14. Before the heart transplantation, WES had been applied to this boy. Interestingly, the *LAMA2* c.8842G > A variant had been identified at the first time among patients with ARVC. However, the patient was absent from any other potential-related variants. So that it is critical to demonstrate the relationship between *LAMA2* and ARVC. Within this research, we enrolled histological section assessment to explore the pieces of evidence on standard ARVC changes. Most important, we reprogrammed the PBMCs of this proband to iPSC. After differentiating iPSC-CMs successfully, bulk RNA-seq was involved to reveal the molecular alternations post *LAMA2* mutation, which would definitely help to underline the association between *LAMA2* and ARVC.

Arrhythmogenic right ventricular cardiomyopathy has been characterized as a heritable disorder with palpitations, syncope, ventricular tachycardia (VT), or fibrillation. Ventricular dysfunction and heart failure can also develop can be found in some patients. This kind of disease was initially considered to be a developmental abnormality of the right ventricle. With the emerging pieces of evidence on molecular diagnosis, a novel genotype-based clinicopathology classification of arrhythmogenic cardiomyopathy (ACM) has been well established. Several specific mutations within desmosome would result in biventricular or only left ventricular phenotype, such as DSP, PLN, and CTNNA3. Besides, large unknown mutations have been claimed to be associated with left ventricular failure, leading to heart transplantation. For this patient, *LAMA2* c.8842C > A could be one of the potential mutations to cause cluster 4 ACM. After the research database, only four reports have been retrieved on *LAMA2* c.8842C > A. Three of them were identified among patients with muscular dystrophy, and only one record demonstrated a potential link with primary dilated cardiomyopathy. In addition, the iPSC-CMs provided a good platform to study the molecular mechanisms due to an uncertain mutation. The bulk RNA-seq results demonstrated alternations among calcium handling, impaired cell-cell interaction, reduced cellular adhesion, and dysfunction of lipid metabolism. All the changes in gene expressions and regulation fall within the pathological remodeling of ARVC. Taking the histological studies of the proband’s abandoned heart, it would draw a highly convinced association between *LAMA2* c.8842C > A and ARVC.

After reviewing all the published cases of *LAMA2* missense mutation, a case series of 57 particular variants was assembled and summarized in Table 1. Most of the mutations were correlated with the symptom of muscular dystrophy, and there was no association with cardiac-related clinical manifestations in theses mutations. Among them, 15 variants were located in the N-terminal domain, 18 variants were located in EGF-like domains, and 16 variants were located in LG domains. But there was no reported pathogenic missense mutation in the LG5 domain.

**TABLE 1:** A summary of reported cases of *LAMA2* missense mutation.

References	Exon	Variants	Predicted amino acid change	Protein domains	Phenotype
Oliveira	1	c.112G > A	p.Gly38Ser	N-terminal	MDC1A
Oliveira	2	c.245A > T	p.Gln82Leu	N-terminal	Late-onset LAMA2-related MD
Ganapathy	2	c.250C > T	p.Arg84Ter	N-terminal	MDC1A
Oliveira	3	c.437C > T	p.Ser146Phe	N-terminal	MDC1A
Gavassini	4	c.454T > G	p.Trp152Gly	N-terminal	LGMD
Geranmayeh	4	c.470C > T	p.Ser157Phe	N-terminal	MDC1A
Di Blasi	4	c.500A > C	p.Gln167Pro	N-terminal	MDC1A
Harris	4	c.611C > T	p.Ser204Phe	N-terminal	LGMD
Dean	5	c.713C > A	p.Ala238Asp	N-terminal	LGMD
Gavassini	5	c.728T > C	p.Leu243Pro	N-terminal	LGMD
Oliveira	5	c.745C > T	p.Arg249Cys	N-terminal	MDC1A
Marques	5	c.812C > T	p.Thr271Ile	N-terminal	Epileptic encephalopathy
Oliveira	5	c.818G > A	p.Arg273Lys	N-terminal	MDC1A
Beytía	6	c.830C > T	p.Ser277Leu	N-terminal	MDC1A
Gavassini	6	c.850G > A	p.Gly284Arg	N-terminal	LGMD
Oliveira	10	c.1326T > G	p.Cys442Trp	EGF-like 3	MDC1A
Töpf	10	c.1466A > G	p.Lys489Arg	EGF-like 4	LGMD
Xiong H	11	c.1553G > A	p.Cys518Tyr	EGF-like 5; first part	MDC1A
Tezak/Valencia	11	c.1580G > A	p.Cys527Tyr	EGF-like 5; first part	MDC1A
Ganapathy	12	c.1749C > G	p.Tyr583Ter	IV type A 1	MDC1A
Beytía	14	c.2089A > G	p.Ile697Val	IV type A 1	MDC1A
Marques	18	c.2461A > C	p.Thr821Pro	EGF-like 7	Epileptic encephalopathy
Xiong H	18	c.2462C > T	p.Thr821Met	EGF-like 7	MDC1A
Tezak	19	c.2584T > C	p.Cys862Arg	EGF-like 7	MDC1A
Allamand	21	c.2954G > A	p.Cys985Tyr	EGF-like 10	MDC1A
Guicheney	21	c.2986T > C	p.Cys996Arg	EGF-like 10	MDC1A
Valencia	22	c.3154A > G	p.Ser1052Gly	EGF-like 11	MDC1A
Töpf	23	c.3235T > C	p.Cys1079Arg	EGF-like 12	LGMD
Oliveira	23	c.3235T > G	p.Cys1079Gly	EGF-like 12	Late-onset LAMA2-related MD
Xiong H	27	c.3931T > G	p.Trp1311Gly	IV type A 2	MDC1A
Ganapathy	27	c.4048C > T	p.Arg1350Ter	IV type A 2	MDC1A
Ganapathy	29	c.4198C > T	p.Arg1400Ter	EGF-like 14; second part	MDC1A
Di Blasi	30	c.4405T > C	p.Cys1469Arg	EGF-like 16	MDC1A
Oliveira	31	c.4523G > A	p.Arg1508Lys	EGF-like 16	MDC1A
Wang	32	c.4640C > T	p.Thr1547Met	EGF-like 17	MDC1A
Oliveira	32	c.4654G > A	p.Ala1552Thr	EGF-like 17	MDC1A
Allamand	32	c.4690C > T	p.His1564Tyr	EGF-like 17	MDC1A
Tezak	33	c.4750G > A	p.Gly1584Ser	Domain II and I	MDC1A
Afshin	33	c.4840A > G	p.Asn1614Asp	Domain II and I	MDC1A
Geranmayeh	38	c.5530C > A	p.Arg1844Ser	Domain II and I	MDC1A
Oliveira	46	c.6548T > G	p.Leu2183Arg	G-like 1	MDC1A
Giugliano	47	c.6599G > A	p.Arg2200His	G-like 1	MDC1A
Töpf	47	c.6624G > C	p.Trp2208Cys	G-like 1	LGMD
Oliveira	47	c.6707G > A	p.Arg2236Lys	G-like 1	LGMD
Töpf	49	c.7040G > T	p.Gly2347Val	G-like 2	LGMD
Ganapathy	50	c.7147C > T	p.Arg2383Ter	G-like 2	MDC1A
Gavassini	52	c.7431A > T	p.Arg2477Ser	G-like 2	LGMD
Oliveira	54	c.7571A > T	p.Glu2524Val	–	MDC1A
Afshin	55	c.7681G > A	p. Gly2561Ser	G-like 3	MDC1A
He	55	c.7691T > C	p.Leu2564Pro	G-like 3	MDC1A
Ganapathy	56	c.7816del	p.Met2606Ter	G-like 3	MDC1A
Geranmayeh	56	c.7881T > G	p.His2627Gln	G-like 3	MDC1A
Xiong H	56	c.7898G > C	p.Gly2633Ala	G-like 3	MDC1A
Magri	60	c.8544C > G	p.His2848Gln	G-like 4	LGMD
Töpf	61	c.8654T > A	p.Leu2885Gln	G-like 4	LGMD
Liang	61	c.8654T > C	p.Leu2885Pro	G-like 4	MDC1A
Fattahi	61	c.8665G > A	p.Gly2889Arg	G-like 4	MDC1A

The laminin-211 structure consists of α2, β1, and γ1 chains. The polymeric activity of laminin-211 maps to three domains of LN: nidogen binds γ 1-Leb3, agrin binds the curl γ1 subunit, integrin α7β1 binds LG1-3, α-dystroglycan (α-DG) binds to LG4-5 with sulfated glycolipids. LG interactions provide anchoring for the cell membrane and cytoskeleton. α-DG is coupled to transmembrane β-DG, β-DG binds to cytoskeletal dystrophin, and dystrophin binds to the actin-rich cytoskeleton; this critical interaction creates a link from the basement membrane to actin cytoskeleton (11). Previous studies demonstrated homozygous or compound heterozygous mutations that result in deletion of the α2 subunit or diverse aggregation of laminin-211 lead to muscular dystrophy with peripheral nerve defects like MDC1A or LGMD.

In this case, the heterozygous mutation was located in the LG5 domain. We used SFIT, PolyPhen-2, and MutationTaster to predict the impact of this mutation on protein structure and function, showing that p.G2948S is a diseasing-causing and protein-damaging mutation. The Western blot showed that the expression of LAMA2 protein decreased in the ARVC group, and the expression of LAMA2-interacting proteins (ITGB1, β-DG) increased as a compensatory regulation. Using bulk RNA-seq, it confirmed that DEGs in iPSC-CMs of the ARVC group were abundant in signaling pathways of inflammation, fibrosis, adipose, cell connection, and apoptosis. Thus, we elucidated that p.G2948S of *LAMA2* induced the change of the LG5 domain and induce the compensatory expression of ITGB1 and β-DG, and led to the activation of ARVC changes. Moreover, the impaired binding function of the LG5 domain would reshape the cytoskeleton, leading cardiac failure as a major clinical concern. There are still some limitations in this study. We did not perform the isogenic iPSC line based on this patient. At the same time, the distribution of LAMA2 by immunostaining of iPSC-CM had not been investigated, which we would carry out further analysis in the following studies.

## Conclusion

In summary, this is the first case report to describe an ARVC phenotype with a *LAMA2* mutation of c.8842G > A (p.G2948S), which is located in the LG5 domain. In addition, the first iPSC cell line has been established based on the proband. Histological studies and bulk RNA-seq have demonstrated a convinced association between *LAMA2* c.8842C > A and ARVC. Considering 35–50% of patients with a clinical diagnosis of ARVC fail to address a particular disease-causing gene mutation, this research expands the understanding of the pathogenesis of ARVC. However, the molecular mechanism of *LAMA2* mutation inducing ARVC still requires further study.

## Ethics statement

The studies involving human participants were reviewed and approved by the Ethics Committee of West China Second Hospital of Sichuan University (2014-034). Written informed consent to participate in this study was provided by the participants’ legal guardian/next of kin. Written informed consent was obtained from the individual(s), and minor(s)’ legal guardian/next of kin, for the publication of any potentially identifiable images or data included in this article.

## Author contributions

SL was the patient’s physician and responsible for the revision of the manuscript for important intellectual content. YW reviewed the literature and contributed to manuscript drafting. YW and YL performed the mutation analysis. DZ and YF contributed to the iPSC-related experiments. YL and SL conceptualized and designed the study, coordinated and supervised the data collection, and critically reviewed the manuscript for important intellectual content. All authors issued final approval for the version to be submitted.

## Conflict of Interest

The authors declare that the research was conducted in the absence of any commercial or financial relationships that could be construed as a potential conflict of interest.

## Publisher’s Note

All claims expressed in this article are solely those of the authors and do not necessarily represent those of their affiliated organizations, or those of the publisher, the editors and the reviewers. Any product that may be evaluated in this article, or claim that may be made by its manufacturer, is not guaranteed or endorsed by the publisher.

## Footnotes

1
https://swissmodel.expasy.org/

2
http://biosig.unimelb.edu.au/mcsm/

3
http://marid.bioc.cam.ac.uk/sdm2

4
http://biosig.unimelb.edu.au/dynamut/

5
http://biosig.unimelb.edu.au/duet/

6
https://www.genome.jp/kegg

## Supplementary Material

Supplementary Figure 1 KEGG enrichment analysis of the fibrosis signaling pathway.

Supplementary Figure 2 KEGG enrichment analysis of the lipidation signaling pathway.

Supplementary Figure 3 KEGG enrichment analysis of the apoptosis signaling pathway.

Supplementary Figure 4 KEGG enrichment analysis of the calcium signaling pathway.
