# Supplementary figures and images for: A rare case of arrhythmogenic right ventricular cardiomyopathy associated with LAMA2 mutation: A case report and literature review
Source: Front Med (Lausanne). 2022 Jul 18;9:922347. doi: 10.3389/fmed.2022.922347 (PMC9339636; doi:10.3389/fmed.2022.922347)

# KEGG enrichment analysis(fibro)

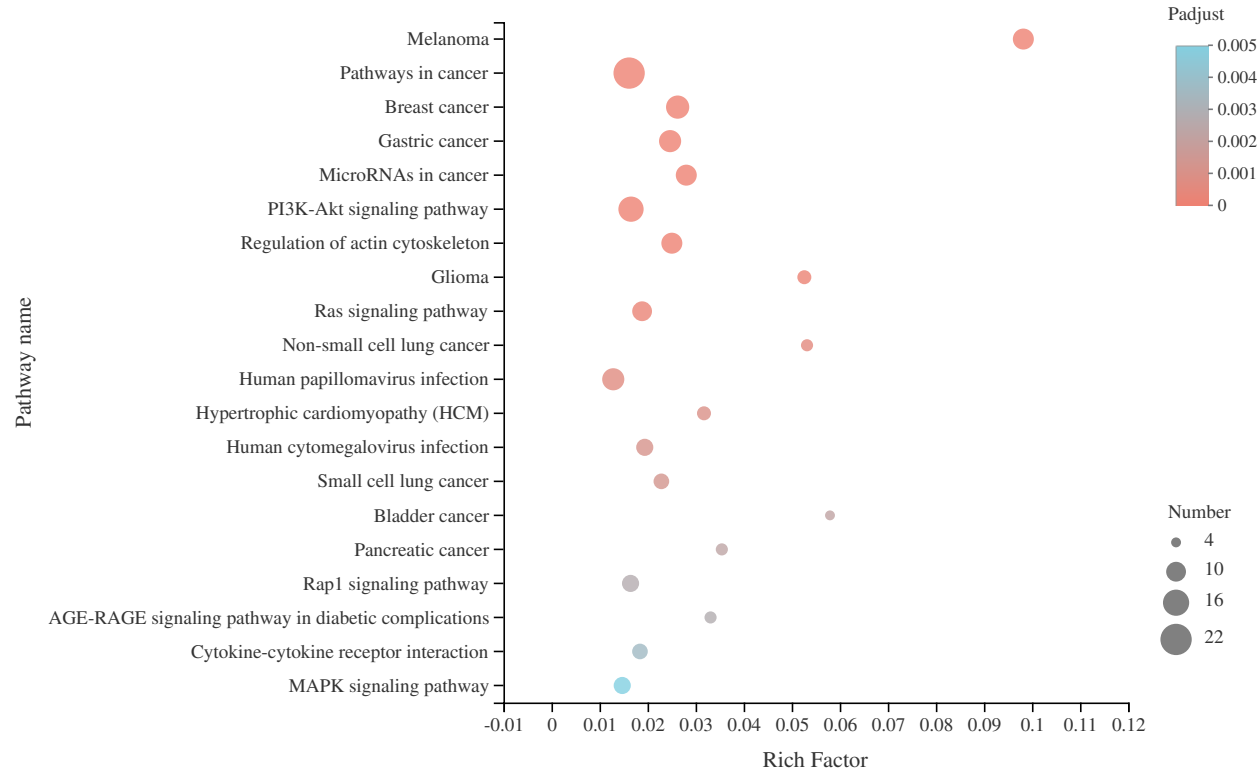

Supplement: Supplementary Figure 1 — KEGG enrichment analysis of the fibrosis signaling pathway. [file Data_Sheet_1.PDF]

# KEGG enrichment analysis(lipid)

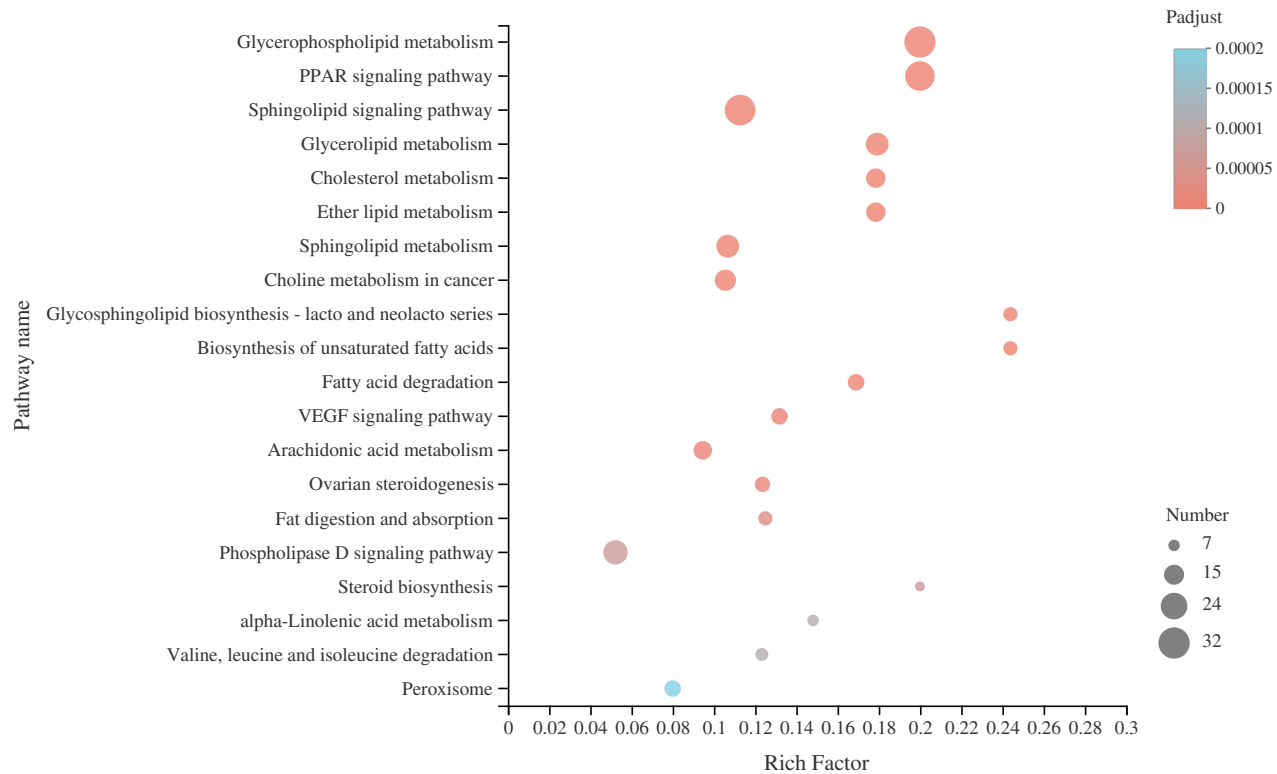

Supplement: Supplementary Figure 2 — KEGG enrichment analysis of the lipidation signaling pathway. [file Data_Sheet_2.PDF]

# KEGG enrichment analysis(apoptosis)

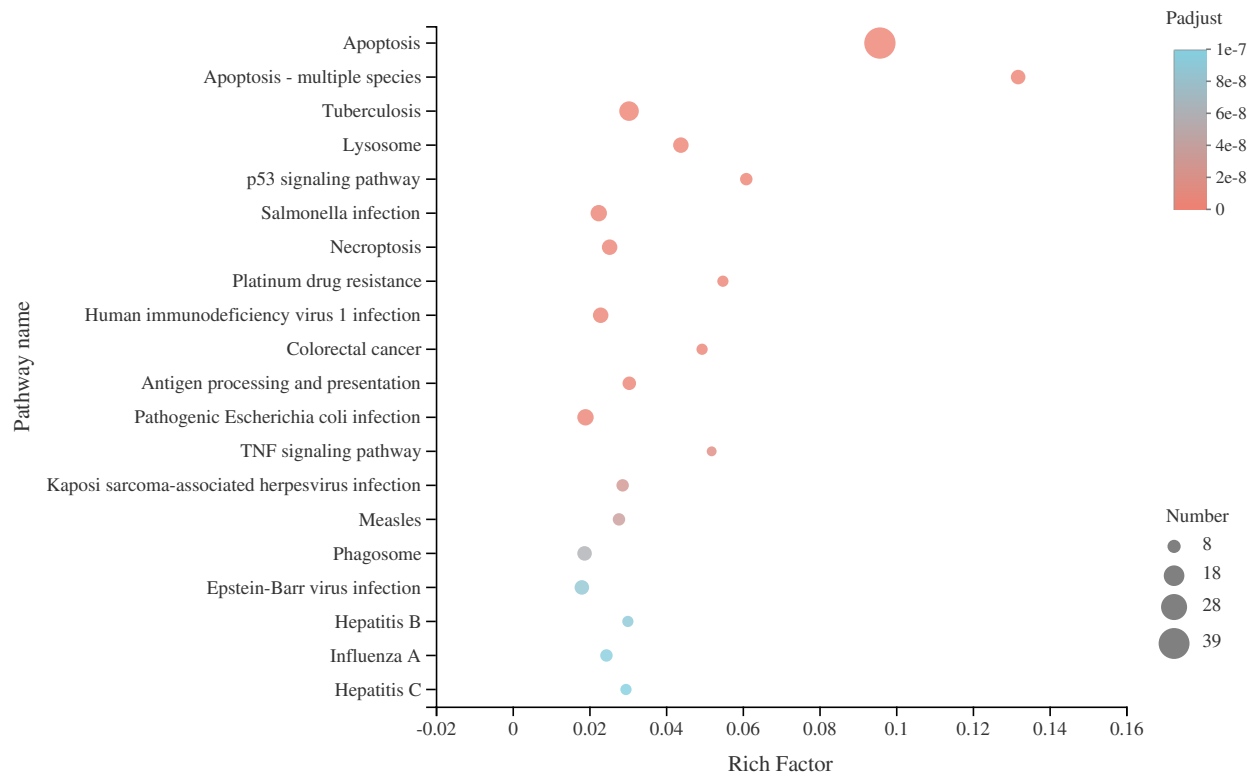

Supplement: Supplementary Figure 3 — KEGG enrichment analysis of the apoptosis signaling pathway. [file Data_Sheet_3.PDF]

# KEGG enrichment analysis(calcium)

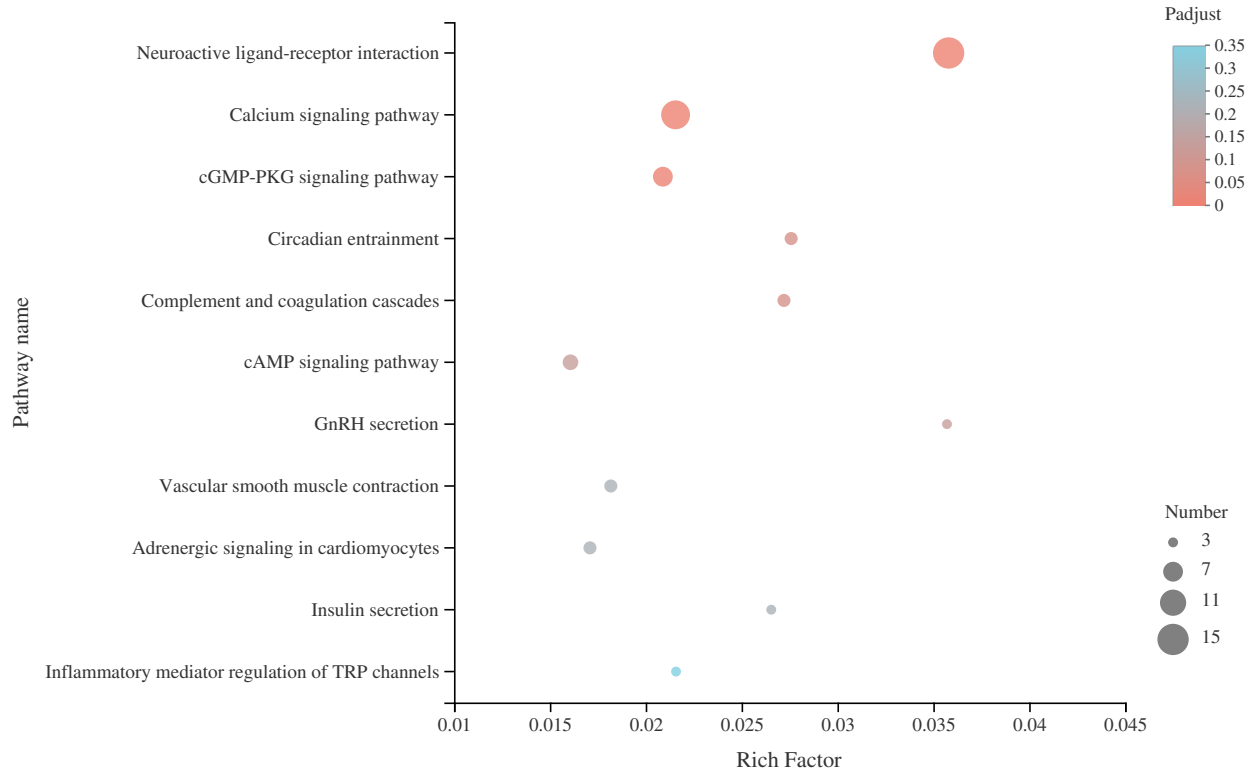

Supplement: Supplementary Figure 4 — KEGG enrichment analysis of the calcium signaling pathway. [file Data_Sheet_4.PDF]
